# Is More Always Better With Digital Health Interventions? Shifting Engagement From Maximizing Use to Supporting Health

**DOI:** 10.1016/j.mcpdig.2026.100368

**Published:** 2026-04-30

**Authors:** Michael P. Williams, Susan A. Murphy, Ana-Maria Vranceanu

**Affiliations:** a Center for Health Outcomes and Interdisciplinary Research, Massachusetts General Hospital, Boston, MA; b Department of Psychiatry, Harvard Medical School, Boston, MA; c Kempner Institute, Harvard University, Boston, MA

Since the early 2000s, research into digital health interventions (DHIs) has expanded dramatically, particularly in “mHealth.” Alongside this growth, scholars have increasingly examined how users engage with these interventions and how engagement can be optimized. Yet, much of this work has equated engagement with frequency or duration of use, seemingly treating maximization of utilization as the ultimate goal. This prevailing focus overlooks a more meaningful dimension of engagement, namely, the quality of users’ interactions with DHIs and their relevance to well-being and behavior change.

## The Diverse Roots and Vague Definitions of Engagement

The term engagement originated in industrial and organizational psychology, coined by Kahn,^1^ to describe how employees became “personally engaged” at work by authentically expressing themselves cognitively, emotionally, and physically. This idea evolved into the construct of “work engagement.” Yet even within this domain, competing definitions proliferated around a fundamental question: is engagement a stable trait or a dynamic relationship between employee and organization?

Concurrently, scholars in computer science and human-computer interaction developed their own definitions of engagement in the context of digital technologies, often drawing from past psychology theory such as play or flow.^2^^,^^3^ Despite differences in theoretical underpinnings and operationalizations, these definitions typically described engagement as a positive immersive experience between a user and a technology. By the mid-2000s, however, marketing agencies and practitioners appropriated the term to describe building deep emotional connections with potential and current customers to drive loyalty and profit. This fusion of engagement as both a pleasurable user experience and a mechanism for commercial gain fundamentally reshaped the concept and its application to digital health into the value-laden construct that it is today.

### Paradigm Shift: From Interaction to Optimization

As organizations recognized the profit potential of sustained user attention, the concept of engagement underwent a profound shift. Once understood as a self-directed relationship between an individual and a technology, engagement has increasingly become something to be engineered, optimized, and even imposed upon users.

For revenue generation, this transformation is economically rational. For example, clothing companies will use targeted campaigns and influencer partnerships that blur the line between authentic recommendation and advertising to maximize customer interaction with their brand.

To support these goals, the digital ecosystem has been deliberately structured to sustain engagement and link it directly to sales channels. Social media platforms, in particular, are designed to keep users active for as long as possible by exploiting psychologically compelling mechanisms such as fear of missing out, algorithmic exposure to emotionally charged or aspirational content, upward social comparison, and even “ragebait” posts engineered to provoke reaction. In this paradigm, engagement is no longer an organic experience but a commercial metric to be maximized.

### Social Media Engagement as the Default Paradigm

The sales-driven, social media–based model of engagement optimization has quietly shaped how we think about engagement across domains, including in digital health research. Many studies now treat participant engagement as an app utilization problem to be solved, implicitly assuming that *more is better.* This mindset permeates the entire research pipeline, from funding priorities to publication norms.

Funders, especially at the feasibility stage, often require investigators to demonstrate that DHIs meet specific “engagement” benchmarks, typically defined as app utilization thresholds such as days logged in or minutes spent online. Although such measures can be useful to flag insufficient use, they inherently fail to capture the *quality* or *meaningfulness* of engagement. Similarly, at the reporting stage, engagement outcomes are often presented as app utilization metrics under the assumption that higher usage automatically produces better health outcomes, a link that remains inconsistently supported by evidence.^4^^,^^5^ Even when studies examine how utilization relates to outcomes, they are limited by design. Participants are randomized to intervention arms rather than utilization dosages, meaning associations are confounded (eg, disease severity and motivation). In contrast, for example, pharmaceutical research historically employs dosing studies to establish relationships between exposure, efficacy, and adverse effects. There is potential for optimization-based experimental designs, such as the MOST (Multiphase Optimization Strategy) framework, to be applied to dosing in digital health.^6^^,^^7^ This framework is intended to estimate causal effects at specific intervention levels on health outcomes, which could be used to develop evidence-based engagement targets. Yet, its application to dose-response questions remains nascent in many domains. As such, a paradigm shift in our conception of digital health engagement is needed.

### Centering a Healthy Lifestyle in DHI Engagement Optimization

As daily life becomes increasingly dominated by digital technologies competing for attention, it is time to redefine *engagement* in DHIs around what truly matters: health. Yardley et al’s^8^ concept of *effective engagement*, defined as “sufficient use needed to achieve health outcomes,” offers a critical corrective.

The guiding principle of DHI design, evaluation, and implementation should be to support each individual’s adoption and maintenance of a sustainable healthy lifestyle. Every microinteraction within a DHI should serve this broader purpose. The goal is not to maximize total app utilization time, but to facilitate lasting behavior change that integrates seamlessly into daily life.

Healthy living on a day-to-day basis is largely defined by *balance*. Almost all health behaviors are practiced in moderation and come with an opportunity cost. Yardley et al^8^ discuss how these trade-offs change throughout the course of DHI-facilitated behavior change. In many contexts, such as healthy eating or smoking cessation, high levels of rigid engagement may be beneficial for most people, especially throughout the early phases of behavior change (eg, logging all food consumed in a diet tracking app). However, it is important to note that time spent meticulously logging every ounce of food one consumes in a diet tracking app comes at the cost of doing something else. As individuals progress, engagement could become progressively more relaxed and dynamic, helping individuals design systems that may or may not include the DHI itself, that can facilitate the maintenance of these behaviors. This allows individuals to integrate these behaviors into their normal life and avoid many potential negative effects of long-term, sustained, rigid app use such as dependency, loss of agency, obsessiveness, or other mental health issues. Finally, this paradigm of engagement cannot fit within the profit-driven paradigm inherited from social media platforms that demand increasingly high levels of app utilization. Moving forward, digital health researchers and designers must center their work on fostering balance, autonomy, and sustainable well-being by treating engagement not as an end in itself, but as a means to living well.

### Recommendations for Digital Health Research and Design

Centering a healthy lifestyle in DHI design and engagement research is an intrinsically transdisciplinary endeavor. Critical perspectives from every discipline with a stake in human flourishing will be necessary to transform digital health into a unified infrastructure of care. Nielsen et al’s^9^ National Institutes of Health Science of Behavior Change Program offers a principled framework for navigating this transformation in digital health engagement. This framework underscores the importance of prespecifying a theoretically informed causal model, including mechanisms of change, in behavior change intervention development. Merging Science of Behavior Change Program with the idea of effective engagement proposed by Yardley et al^8^ provides a provisional framework for constructing causal models in digital health (see the Figure).

**Figure:**
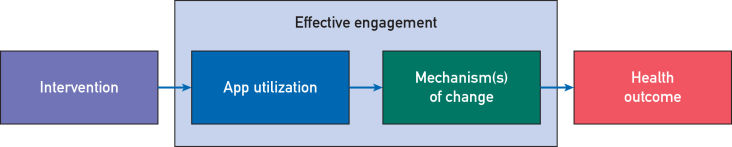
Provisional framework integrating science of behavior change and effective engagement.

In this framework, DHIs should motivate app utilization that is linked to changes in key mechanisms (ie, effective engagement), which subsequently drives changes in target health outcomes. From this framework, it is possible to offer concrete guidelines for the design, evaluation, and refinement of theoretically informed DHIs (Table). Importantly, this framework supports evaluation of DHIs both as discrete interventions and as features embedded in the context of one’s life.

**Table:** Guidelines for Design and Research for Engagement in Digital Health

Component	Recommendations
Intervention	• Designed for prespecified, theory-informed engagement pattern • Attention, time, and interaction pattern-matched control arm • Intervention components specified in terms of discrete options available at defined decision points • Use formative research to identify population-specific characteristics that inform personalization and drive engagement • Educate participants on engagement dosing and behavior change
Utilization	• Operationalize utilization across multiple dimensions (eg, effort, burden, and acceptability) • Continual monitoring of near-time measures of burden and adherence through use of novel tech (eg, wearables, passive sensing, and ecological momentary assessments) • Scheduled monitoring of intermediate-time measures of burden, perceived utility, and acceptability (eg, weekly) • Use optimization and dose-finding studies to establish minimum and maximum effective utilization thresholds for producing mechanism activation
Mechanism(s) of change	• Specify mechanisms of change as the proximal intervention target • Align mechanism measure selection and timing with engagement pattern • Continual monitoring near-time proximal mechanisms • Scheduled monitoring of mechanisms (eg, weekly or monthly)
Health outcome	• Proximal health outcomes evaluated through the lens of surrogate outcomes (eg, daily step count → cardiometabolic risk) • Distal health outcomes measured at postintervention and long-term follow-up using measures optimized for precision, validity, and sensitivity to intervention-induced change
Overall	• Theory-informed components, including selection and design of measures, intervention components, and engagement pattern • Characterize and statistically test associations between framework components

When designing a novel DHI, intervention components should be derived from established domain-relevant theories. For example, P3 (Prepared, Protected, emPowered), a pre-exposure prophylaxis adherence intervention for adolescents and young adults at risk for HIV, modeled intervention components after social cognitive theory.^10^^,^^11^ P3 included adherence coaches and tailored messages from experts who modeled adherence for participants, enacting a fundamental aspect of social cognitive theory: vicarious experiences. Further, intervention components should be prespecified in terms of discrete options that are delivered at defined decision points. For example, a future trial of P3 may define a decision point at the daily medication administration window, with discrete options including a reminder notification, a motivational message from a coach, or no contact. Further, eligibility criteria themselves can be conceptualized as an initial, fixed decision point as this determines who receives the intervention at all. Clearly defining all decision points enables transparency in reporting and comparison with the theoretical model underlying the intervention.

In addition, DHI design should be a process of co-design between designers and the people the DHI is built to serve. As DHIs evolve from static websites to adaptive, data-driven systems (eg, just-in-time adaptive interventions, which dynamically tailor content and delivery timing based on user context), researchers have an unprecedented opportunity to cocreate digital health experiences with participants. These intervention design paradigms hold great potential for shifting engagement from an app utilization metric to be maximized toward a collaborative process that fosters meaningful, individualized health behavior change.

Moreover, careful consideration for the research paradigms in which we evaluate DHIs is warranted. Formative research methods (ie, National Institutes of Health stage 1 methods),^12^ such as interview studies and feasibility pilot studies, are instructive for identifying areas for personalization and tailoring. Further, control conditions in randomized controlled trials of DHIs should be matched to the intervention on time, attention, and interaction pattern. For example, if a DHI largely relies on audio-recorded content for the participants to listen to, the control arm should also use audio-recordings that are delivered at roughly the same dose and frequency. Finally, as DHIs continue to evolve into more advanced context-aware and multicomponent designs, it will become increasingly necessary to use optimization-based experimental designs (eg, MOST, SMART [Sequential Multiple Assignment Randomized Trial], and microrandomized) to establish which components work, for whom, at what time, and at what dose.^6^^,^^7^^,^^13^^,^^14^

App utilization should be studied as a multifaceted construct (eg, burden, effort, and acceptability), as opposed to solely in terms of utilization volume. Further, utilization captures only one-half of effective engagement. Utilization and interaction patterns should be designed to target mechanisms of change, and measurement of utilization and mechanisms should be theory-driven and mutually aligned. For example, if provision of social support is a putative mechanism of change, then utilization of social support should be explicitly characterized (eg, messages sent/received and coding social interactions), and validated measures of social support should be administered to measure changes. In modern DHI settings, designers should use log data and passive sensing technologies to enable continual monitoring of near-time measures of burden, app utilization, adherence to nondigital behaviors (eg, medication adherence), and mechanisms (eg, step count as a proxy for physical activity). In addition, intermediate-time measures of burden, perceived utility, acceptability, and mechanisms of change should be scheduled and monitored on a regular basis (eg, weekly) to ensure that the DHI and engagement pattern are moving the intended targets. The systematic collection of these data is required for optimization-based experimental designs needed to establish evidence-based guidelines for engagement that move beyond utilization benchmarks.

Finally, measurement of proximal and distal health outcomes should be delineated and prespecified. For proximal health outcomes, surrogate health outcomes (ie, intermediate outcomes that are used as substitutes for clinical outcomes of interest)^15^ should be considered as a way to balance feasibility and precision. Because participant burden is often significantly lower for measuring surrogate health outcomes, measures can be collected at a higher frequency and used to guide decision-making. Conversely, distal health outcomes should be collected at postintervention and follow-up assessments and optimized for precision, validity, and sensitivity to intervention-induced change, as these measures capture movement in the final target of the intervention.

## Conclusion

This commentary tracks how the paradigm of engagement for DHIs has unconsciously inherited a profit-motivated disposition and argues that engagement in DHIs needs to be recentered around balanced, healthy living. We propose a provisional framework and discuss implications for the design and testing of DHIs. Engagement in digital health is ultimately a question of values, and those values should be centered on technology that enables a balanced lifestyle for the people it aims to serve.

## Potential Competing Interests

The authors report no competing interests.
